# Next-generation CsPbBr_3_ perovskite nanocrystal chloride sensors: stability engineering and halide-exchange mechanisms in aqueous, biological, and environmental media

**DOI:** 10.1039/d5ra09798c

**Published:** 2026-05-20

**Authors:** Mohamed Abu Shuheil, Ahmed Aldulaimi, Subhashree Ray, Talal Aziz Qassem, Gunjan Garg, Renu Sharma, Dilbar Urazbaeva, Sabokhat Sadikova, Sharmin Smaeilpour

**Affiliations:** a Faculty of Allied Medical Sciences, Hourani Center for Applied Scientific Research, Al-Ahliyya Amman University Amman Jordan; b Faculty of Pharmacy, Al-Zahrawi University Karbala Iraq; c Department of Biochemistry, IMS and SUM Hospital, Siksha ‘O’ Anusandhan (Deemed to be University) Bhubaneswar Odisha-751003 India; d Department of Medical Laboratory Technics, College of Health and Medical Technology, Alnoor University Mosul Iraq; e Centre for Research Impact & Outcome, Chitkara University Institute of Engineering and Technology, Chitkara University Rajpura Punjab 140401 India; f Department of Chemistry, University Institute of Sciences, Chandigarh University Mohali Punjab India drrenusharma01@outlook.com; g Department of Psychology and Medicine, Mamun University Khiva Uzbekistan; h Department of Chemistry, Urgench State University 220100 Urgench Uzbekistan; i Young Researchers and Elite Club, Tehran Branch, Islamic Azad University Tehran Iran sharminsmaeilpour@gmail.com

## Abstract

This review provides a comprehensive overview of next-generation Cesium lead bromide (CsPbBr_3_) perovskite nanocrystals (PNCs) for chloride ion sensing, with a focus on stability engineering and halide-exchange mechanisms. The article summarizes recent advances in structural and surface-engineering strategies—including core–shell architectures, polymer and inorganic encapsulation, compact surface ligands, and compositional modifications—that have been developed to enhance the environmental and chemical stability of CsPbBr_3_ PNCs in aqueous and complex media. In parallel, the fundamental principles governing halide exchange are described through established relationships such as lattice contraction, bandgap bowing, photoluminescence–composition correlations, exchange kinetics, and equilibrium behavior. These theoretical foundations are linked to the optical response of CsPbBr_3_-based chloride sensors and their fast, reversible spectral shifts. Furthermore, studies employing CsPbBr_3_ PNCs in aqueous, biological, and vapor environments are summarized, highlighting opportunities as well as common limitations related to stability, selectivity, and operational durability. Overall, this review consolidates current knowledge on engineering approaches and mechanistic understanding, providing a unified perspective on the design and performance of CsPbBr_3_ nanocrystal-based chloride sensing platforms.

## Abstract 2

Stability-engineered CsPbBr_3_ nanocrystals enable reversible chloride sensing through Br^−^/Cl^−^ exchange, causing lattice contraction and a predictable green-to-blue photoluminescence shift for portable optical detection.

## Graphical abstract

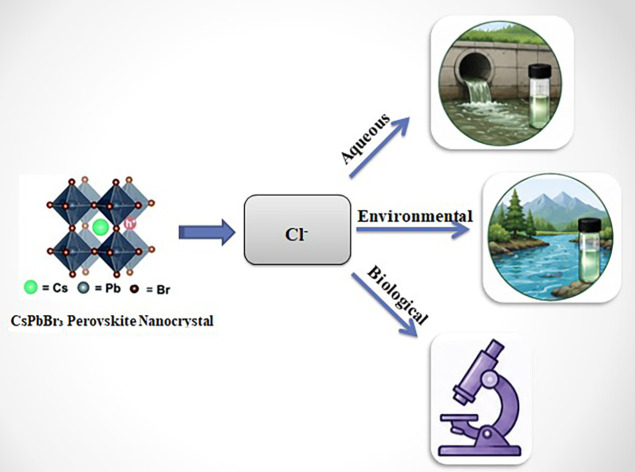

## 1. Introduction

In recent years, halide perovskite nanocrystals (PNCs)—particularly cesium lead bromide (CsPbBr_3_)—have emerged as one of the most transformative classes of materials in optoelectronics due to their exceptional photoluminescence quantum yields (PLQY), narrow emission linewidths, and highly tunable bandgaps.^1–3^ Beyond their established roles in light-emitting diodes, lasers, and photodetectors, these nanocrystals have opened a rapidly expanding avenue in chemical and ionic sensing. Among various targets, chloride ion detection has attracted significant attention owing to its critical importance in environmental monitoring, industrial process control, and biomedical diagnostics. Traditional chloride determination methods, including electrochemical probes, ion chromatography, and colorimetric assays, often require labor-intensive procedures, trained personnel, or sophisticated instrumentation. In contrast, CsPbBr_3_ nanocrystals offer a uniquely fast, optical, and visually trackable response derived from their intrinsic halide-exchange properties, positioning them as highly promising candidates for next-generation chloride sensors.^4–7^

The hallmark of CsPbBr_3_ nanocrystals lies in their unusually soft ionic lattice, where low migration barriers enable rapid anion diffusion. Exposure to chloride ions triggers a spontaneous Br^−^ → Cl^−^ substitution within the perovskite framework, leading to a predictable blue-shift in both absorption and photoluminescence (PL) spectra.^8,9^ This exchange occurs on timescales of milliseconds to seconds, an order of magnitude faster than in conventional semiconductor nanomaterials. Such ultrafast kinetics stem from the highly dynamic nature of the perovskite lattice, characterized by shallow defect energies, high ionic conductivity, and pronounced lattice polarizability.^10,11^ These properties enable chloride sensing without the need for external activators, signal amplifiers, or complex surface chemistries. The simplicity, speed, and linear tunability of the optical response provide a foundation for designing sensors that are not only sensitive and selective also cost-effective.^12,13^

Despite these advantages, the practical translation of CsPbBr_3_ nanocrystal-based chloride sensors faces substantial challenges. The intrinsic instability of lead halide perovskites in water, humidity, and polar environments remains the most prominent barrier to widespread adoption. CsPbBr_3_ nanocrystals readily undergo hydration, surface ligand desorption, and structural decomposition when exposed to aqueous media or high ionic strength conditions.^14,15^ These degradation pathways compromise luminescence, distort halide-exchange dynamics, and ultimately limit the operational lifetime of the sensors. Addressing these limitations requires innovative stability engineering strategies capable of preserving structural integrity while maintaining rapid anion accessibility—a demanding balance that has shaped the direction of contemporary research.^16–18^

Recent advances have introduced multiple architectural and chemical approaches to enhance the environmental durability of CsPbBr_3_ nanocrystals. Core–shell structures such as CsPbBr_3_@CsPb_2_Br_5_ or hybrid organic–inorganic coatings create protective barriers that suppress water penetration while retaining partial ion permeability.^18,19^ Polymer encapsulation strategies—including cross-linked amphiphilic matrices, hydrogen-bond-stabilized networks, and stimuli-responsive shells—offer further stabilization by mitigating ligand detachment and shielding the nanocrystals from aggressive media. Inorganic hosts such as mesoporous silica or titania provide rigid, chemically inert frameworks that physically isolate the nanocrystals while enabling controlled diffusion of chloride ions. Collectively, these engineering solutions have significantly improved sensor durability, allowing CsPbBr_3_ nanocrystals to function in increasingly complex environments, ranging from seawater to biological fluids.^20,21^

Beyond stability enhancement, a deeper understanding of the mechanistic foundations of halide exchange has become essential for designing highly reliable and quantitative sensors. Halide exchange in CsPbBr_3_ is governed by a combination of lattice contraction effects, bandgap bowing, Vegard-type compositional relationships, and diffusion-driven kinetics.^22,23^ Recent theoretical and experimental studies have elucidated the roles of defect chemistry, ligand–surface interactions, and microenvironmental factors in modulating exchange rates and equilibrium compositions. These insights have paved the way for more accurate predictive models that correlate spectral shifts with chloride concentration, enabling precise calibration in both simple and complex matrices. Such mechanistic clarity is crucial for minimizing false positives, improving selectivity against competing ions, and ensuring reproducibility under diverse sensing conditions.^24,25^

A key objective of this review is to convert mechanistic understanding into predictive capability. Rather than treating spectral shifts as isolated phenomenological observations, we organize the literature to show how compositional change, defect-mediated diffusion, ligand–surface equilibria, and microenvironmental constraints can be used to anticipate sensor behavior under different operating conditions. This enables a more quantitative interpretation of chloride-dependent optical response and provides a basis for rational optimization of calibration range, selectivity window, and durability.

The integration of CsPbBr_3_ nanocrystals into practical sensing platforms has expanded rapidly. Colorimetric strips,^26,27^ cellulose-based composites,^28,29^ microfluidic devices,^30,31^ and portable fluorimetric^32,33^ readers now incorporate PNCs to provide real-time chloride detection with minimal sample preparation. The vivid color transitions—from bright green to deep blue—enable naked-eye observation, while high PL intensities ensure strong analytical contrast even at low chloride concentrations. Emerging device architectures are increasingly supported by machine-learning-assisted calibration, smartphone imaging, and automated spectral analysis, moving these nanosensors closer to real-world application in environmental monitoring, industrial diagnostics, and point-of-care testing.^34,35^

Despite remarkable progress, significant opportunities and unresolved challenges remain. Achieving long-term stability under operational conditions, maintaining selectivity in biologically and chemically complex matrices, and addressing environmental concerns associated with lead content are key priorities for future research.^3,29^ The development of encapsulation strategies, scalable synthesis routes, and lead-reduced or lead-free alternatives may play decisive roles in enabling the commercial adoption of perovskite-based chloride sensors.

Although several review articles have addressed perovskite nanocrystals in chem/bio sensing applications,^91–93^ chloride detection using CsPbBr_3_ nanocrystals,^94^ and the broader role of halide ions in nanocrystal chemistry,^95^ these works generally examine sensing performance, material synthesis, or halide chemistry as separate domains. In contrast, this review establishes an integrated physicochemical framework that systematically connects halide exchange thermodynamics and kinetics, defect chemistry, surface and lattice stability engineering, and analytical sensing performance within a unified structure–exchange–stability–response relationship.

The novelty of this framework lies in its predictive integration across scales. Prior reviews have typically discussed halide exchange, nanocrystal stability, or sensing performance as related but largely separate topics. Here, these elements are explicitly linked in a cause-and-effect sequence: structure and surface chemistry determine ion-accessibility and defect population; these factors regulate exchange kinetics and equilibrium behavior; and these exchange characteristics then define sensing outputs such as response speed, spectral resolution, reversibility, selectivity, and operational lifetime. This integrated view enables the formulation of practical design rules—for example, identifying when increased passivation improves durability at the cost of slower ion transport, or when defect-rich interfaces enhance sensitivity but compromise reversibility. Accordingly, the framework is intended not only as a synthesis of current knowledge, but also as a guide for predicting performance trade-offs in future sensor designs.

Rather than treating halide exchange solely as a compositional color-tuning effect, we interpret it as a dynamic ion-migration and vacancy-mediated process governed by lattice softness, surface ligand equilibria, and interfacial diffusion barriers. By explicitly correlating exchange mechanisms with stabilization strategies and their impact on sensitivity, reversibility, selectivity, and operational durability in aqueous, biological, and vapor environments, this review provides a design-oriented synthesis that extends beyond existing summaries and offers predictive guidelines for engineering CsPbBr_3_-based chloride sensing platforms.

Importantly, the aim of this review is not only to summarize the existing literature on halide exchange and stabilization strategies, but to define a predictive conceptual framework for chloride sensing. Specifically, we identify how structural and interfacial descriptors—such as lattice softness, vacancy density, ligand binding dynamics, and diffusion barriers–govern exchange kinetics and thermodynamics, and how these in turn determine analytical figures of merit including sensitivity, selectivity, reversibility, and long-term operational stability. In this sense, the review moves beyond a descriptive synthesis by providing a mechanistically organized basis for sensor design and materials selection.

Over the past decade, authoritative reviews have systematically examined ion and anion exchange processes in halide perovskites from nanocrystals to bulk materials,^96,98^ structural and surface transformations in Cs–Pb–X nanocrystals,^97^ and interface engineering strategies for improving efficiency and operational stability in perovskite-based optoelectronic devices.^99,100^ In parallel, multiple reviews have addressed perovskite nanomaterials in chem/bio sensing platforms^91–93^ and chloride detection using CsPbBr_3_ nanocrystals.^94^ However, these studies largely treat halide exchange chemistry, structural metamorphosis, stability engineering, and sensing performance as distinct research domains. The present review differentiates itself by explicitly integrating these perspectives into a unified mechanistic framework, in which halide exchange is interpreted as a defect-mediated, surface-governed ion-transport process intrinsically coupled to lattice stability, interfacial energetics, and environmental. By systematically correlating exchange kinetics and thermodynamics with stabilization strategies and their direct implications for chloride sensing sensitivity, selectivity, reversibility, and durability, this work provides a cross-disciplinary synthesis that bridges nanocrystal chemistry, stability engineering, and analytical sensor design. To clearly position the present review within the existing literature, Table 1 provides a structured comparison of prior authoritative reviews in terms of scope, focus, and limitations, highlighting the specific conceptual and mechanistic gap addressed herein.

**Table 1:** Comparative positioning of prior reviews (ref. 91–100) and the scope of the present work

Main focus	Scope	Limitation relative to this review	Gap addressed here	Ref.
Perovskite nanomaterials in chemical sensing	General chemo/biosensing platforms	No mechanistic focus on halide exchange-driven sensing	Mechanistic linkage between halide exchange and chloride sensing	91
PNCs in chem/bio sensing	Stability challenges in sensing	Focus on application strategies, not exchange physics	Integration of ion migration and sensing response	92
CsPbX_3_ and composites in sensing	Broad sensing utilities	Descriptive performance summary	Structure–exchange–stability correlation	93
CsPbBr_3_ for chloride detection	Visual Cl^−^ sensing strategies	Limited physicochemical analysis of exchange kinetics	Defect-mediated exchange–response framework	94
Role of halide ions in nanocrystal chemistry	Surface chemistry and growth	Not focused on sensing applications	Translation of halide chemistry into analytical design rules	95
Ion exchange in halide perovskites	Mechanisms across scales	Device-oriented, not sensing-oriented	Application of exchange thermodynamics to sensor	96
Structural metamorphoses of Cs–Pb–X NCs	Transformation chemistry	No analytical sensing context	Coupling metamorphosis pathways to sensing stability	97
Anion exchange processes	Methods and optoelectronic applications	Focus on bandgap tuning	Linking exchange control to selectivity and reversibility	98
Interface engineering in PSCs	Efficiency and stability	Device-level engineering	Adapting stability strategies to colloidal sensing systems	99
Stability engineering in PSC interfaces	Long-term operational durability	Solar cell perspective	Translating interface principles to aqueous chloride sensing	100

While previous reviews have addressed halide exchange mechanisms, optical bandgap modulation, and sensing applications in partially overlapping contexts,^91–98^ the present work aims to provide a quantitatively unified interpretation that explicitly correlates exchange thermodynamics and kinetics, lattice stability, and analytical sensing performance. Rather than introducing these elements independently, this review emphasizes their mechanistic interdependence within chloride-responsive CsPbBr_3_ nanocrystal systems.

## 2. Advanced engineering strategies for stabilizing lead halide PNCs in aqueous media

### 2.1. Epitaxial core–shell growth and Quasi-2D perovskite barrier integration

The Achilles' heel of CsPbBr_3_ nanocrystals is their low formation energy (≈0.1–0.2 eV per formula unit), which makes the lattice highly susceptible to nucleophilic attack by water or hydroxide ions. Epitaxial core–shell engineering directly addresses this by surrounding the emissive core with a chemically more inert, wider-bandgap perovskite phase that shares the same crystal symmetry and very similar lattice constants.^36,37^ Typical shells such as CsPb_2_Br_5_, Cs_4_PbBr_6_, or CsPbBr_3_@CsPbCl_3_-rich gradients are grown in one-pot reactions by injecting excess PbBr_2_ or Cs-oleate at lower temperature after core formation. The resulting type-I band alignment confines excitons within the core while the shell dramatically reduces the surface energy and blocks direct solvent access.^38^

Quasi-2D Ruddlesden–Popper phases (*e.g.*, (BA)_2_PbBr_4_, (PEA)_2_CsPb_2_Br_7_) are increasingly used as outer barriers because the long organic cations create hydrophobic van der Waals gaps that act as natural moisture repellents. These 2D/3D heterojunctions have shown retention of >85% PL intensity after 90 days in distilled water and >60% in artificial seawater. Importantly, these inorganic/hybrid shells do not form an impermeable wall; instead, they function as semipermeable membranes that slow down unwanted water diffusion by 3–5 orders of magnitude while still permitting halide anions to reach the core on a timescale of seconds to minutes—ideal for practical sensing.^39,40^ This strategy has evolved toward graded-composition shells and triple-shell architectures (CsPbBr_3_@CsPb_2_Br_5_@2D-RP), offering simultaneous protection against humidity, light, heat, and ionic strength variations encountered in real-world chloride monitoring scenarios.

### 2.2. Cross-linked amphiphilic polymer networks and stimuli-responsive coatings

Simple physical adsorption of polymers is insufficient for long-term aqueous use. Modern encapsulation employs covalent or strong coordinative cross-linking to create an interpenetrating network that mechanically locks the nanocrystal in place. Polymers containing multiple maleic anhydride, succinimide, or isocyanate groups react *in situ* with surface amines or carboxylates, forming amide or urea bridges. A second generation uses clickable amphiphilic polymers (thiol- or azide-functionalized) that undergo UV- or thermally-triggered thiol–ene or copper-free click chemistry around pre-formed nanocrystals. The resulting covalently stitched shell swells <5% in water and withstands sonication, centrifugation, and filtration without ligand loss.^41–43^

Stimuli-responsive polymers add an extra layer of control: pH-sensitive poly(acrylic acid) derivatives shrink at low pH, temporarily closing ion channels and protecting the perovskite during storage in acidic wastewater, then open again at neutral pH for measurement. Temperature-responsive poly(*N*-isopropylacrylamide) coatings perform the opposite, enabling “on-demand” sensing windows. These smart coatings also incorporate antifouling segments (zwitterionic or polyethylene glycol blocks) that minimize biofouling in sweat or urine matrices. Recent reports show devices retaining full response amplitude after 30 days of continuous immersion in human serum—previously unthinkable for uncoated perovskites.^44,45^ The combination of covalent cross-linking and stimuli-responsivity transforms the nanocrystal from a fragile laboratory curiosity into a reusable sensing platform suitable for wearable or implantable chloride detection.

However, excessive cross-linking density or thick polymer shells can reduce segmental flexibility and partially block ion transport pathways. In such cases, halide exchange kinetics may shift from diffusion-limited to barrier-limited regimes, leading to slower response times. Therefore, polymer encapsulation must be carefully engineered to balance mechanical with sufficient ionic permeability.

### 2.3. Mesoporous inorganic oxide matrices and atomic layer deposition barriers

Embedding CsPbBr_3_ nanocrystals inside continuous mesoporous silica, alumina, or titania matrices creates a “ship-in-a-bottle” structure where the perovskite is physically trapped inside 5–50 nm cages. Synthesis routes include supercritical CO_2_ infusion, non-hydrolytic sol–gel in benzyl alcohol, and microemulsion templating. The oxide framework is optically transparent across UV-Vis-NIR, thermally stable >400 °C, and chemically inert from pH 0–14. Water vapor transmission rates drop below 10^−7^ g m per day, effectively isolating the perovskite from liquid water for years under ambient conditions.^46,47^

Controlled mesoporosity (2–10 nm) ensures rapid chloride diffusion (response <10 s) while larger biomolecules and polyelectrolytes are size-excluded. Surface silanol groups can be further functionalized with alkyl or fluorinated chains to tune hydrophobicity and anion affinity. Atomic layer deposition of 1–8 nm Al_2_O_3_, HfO_2_, or ZrO_2_ over pre-coated silica provides the ultimate barrier: these films are pinhole-free and conformal even on high-aspect-ratio surfaces. Combined SiO_2_–ALD shells have survived autoclaving (121 °C, 2 bar) with <10% PL loss.^48^ Such ultra-composites are now being integrated into optical fibers and microfluidic channels for continuous, maintenance-free chloride monitoring in harsh industrial or marine environments.

While ALD-grown oxide layers provide unparalleled moisture resistance, dense films exceeding several nanometers may significantly suppress halide diffusion. As a result, ALD barriers are most effective for sensing applications only when combined with mesoporous scaffolds or limited to ultrathin (<3–4 nm) coatings that retain controlled ionic accessibility.

### 2.4. Compact zwitterionic, crown-ether, and fluorocarbon surface ligands

The primary weakness of native oleate/oleylamine ligands is their dynamic binding and easy protonation/deprotonation in water. Compact zwitterionic molecules replace them entirely and establish a 0.7–1 nm thick hydration shield that exerts an osmotic pressure of several hundred atmospheres against incoming water clusters, yet presents almost zero energy barrier to small, poorly hydrated anions such as Cl^−^. Crown-ether-type macrocycles (12-crown-4 for Cs^+^, dibenzo-18-crown-6 derivatives for surface-exposed Pb^2+^) anchor tightly to the perovskite surface through multiple oxygen–metal contacts. This local chelation drastically lowers the concentration of mobile cation vacancies, which are the rate-limiting step in hydrolysis and PbBr_2_ leaching.^49–52^

Perfluorinated carboxylates and phosphonates (*e.g.*, perfluorooctanoic acid, perfluorodecyltriethoxysilane, Zonyl FSA) generate ultralow surface energy (<10 mN m^−1^) and induce Cassie–Baxter wetting states even in microliter droplets, trapping stable air plastrons that physically separate the nanocrystal from liquid water for hours. The short ligand length (1–1.5 nm) ensures that >70% of the original surface remains sterically accessible, preserving diffusion-limited anion-exchange rates below 2 seconds while completely eliminating long-term PL decay in 100% water or artificial sweat. Latest designs incorporate photopolymerizable diacetylene or cinnamate tails on zwitterionic heads, enabling secondary UV-induced topochemical polymerization into covalently linked thin films or paper-integrated test strips that retain full halide sensitivity and can be stored dry for over a year without degradation.^53,54^

Compared to polymeric or oxide shells, compact zwitterionic and fluorinated ligands impose minimal diffusion resistance, explaining their superior performance in applications requiring sub-second halide exchange.

### 2.5. Lattice hardening *via* heterovalent doping and mixed-cation/anion compositional engineering

Heterovalent B-site substitution with smaller, more electronegative cations (Mn^2+^, Zn^2+^, Cd^2+^, Sn^4+^, Bi^3+^, Sb^3+^) increases the average metal–halide bond covalency and raises the defect formation energy from ∼0.2 eV to >0.8 eV, making spontaneous vacancy creation thermodynamically unfavorable even at elevated temperature or in polar media. A-site mixing with Rb^+^ or K^+^ contracts the unit cell and pushes the tolerance factor closer to the ideal cubic range (0.9–1.0), suppressing the reversible transformation into hydrated non-emissive phases that normally occurs within minutes in pure water.^55^

Incorporation of pseudohalides (SCN^−^, BF_4_^−^, OCN^−^) or larger halides (I^−^) on the X-sites introduces localized lattice strain fields that act as kinetic traps, dramatically slowing complete anion replacement and preventing the emission from disappearing at very high chloride concentrations. Post-synthetic defect-healing protocols use dry halide-salt vapors (CsCl, ZnBr_2_) or short-wavelength photon soaking refill bromide vacancies and eliminate deep trap states responsible for non-radiative losses after partial Cl^−^ incorporation, routinely pushing PLQY back above 90%.^56,57^ Because no external shell is required, these hardened compositions remain colloidally stable without additional ligands, are fully biocompatible, and can be directly incorporated into edible or implantable formats—critical for future *in vivo* chloride tracking in cystic fibrosis patients or real-time sweat analysis during exercise—while still delivering a clear, monotonic 60–100 nm blue-shift upon chloride exposure.

### 2.6. Comparative trade-offs between stabilization and halide exchange kinetics

Although a wide range of stabilization strategies has been developed for aqueous-compatible CsPbBr_3_ nanocrystals, their impact on halide exchange kinetics varies substantially. A critical comparison reveals that not all stabilization approaches are equally compatible with sensing applications based on fast ion diffusion.

Strategies based on compact surface ligands, such as zwitterionic, crown-ether, and short fluorocarbon molecules, largely preserve diffusion-limited halide exchange. Their sub-nanometer thickness and weak steric hindrance maintain open diffusion pathways while suppressing water-induced degradation. Similarly, quasi-2D perovskite barriers and graded epitaxial shells function as semipermeable membranes: they reduce water permeability by orders of magnitude yet allow small halide anions to reach the core on timescales compatible with real-time sensing.

In contrast, dense cross-linked polymer networks and fully conformal oxide shells prioritize long-term environmental stability at the expense of kinetic accessibility. Highly cross-linked polymers may restrict segmental motion and narrow ion transport channels, leading to slower response times or reduced sensitivity. Likewise, ultradense ALD-grown oxide layers can significantly suppress halide diffusion unless engineered to be ultrathin or combined with mesoporous scaffolds.

Mesoporous oxide matrices represent an intermediate case, offering physical protection while preserving rapid halide exchange through size-selective pore networks. Overall, optimal sensing performance emerges not from maximal encapsulation, but from controlled permeability that balances environmental durability with fast ion transport.

### 2.7. Scalability and manufacturability considerations for real-world deployment

While advanced encapsulation strategies offer exceptional stability, their feasibility for large-scale deployment varies significantly. Techniques such as atomic layer deposition provide unmatched conformality and barrier quality but remain limited by high capital cost, low throughput, and batch-processing constraints. As a result, ALD-based stabilization is currently best suited for high-value applications such as optical fibers, microfluidic sensors, or niche industrial monitoring devices rather than disposable platforms.

Mesoporous oxide matrices offer greater scalability, as sol–gel and templated synthesis routes are compatible with batch and roll-to-roll processing. However, precise control over pore size and uniform nanocrystal loading remains a manufacturing challenge at scale. In contrast, ligand-based stabilization and polymer encapsulation are inherently scalable, relying on solution-phase chemistry compatible with printing, coating, and fiber-spinning technologies.

From a manufacturability perspective, hybrid strategies—such as compact ligands combined with thin inorganic barriers or mesoporous supports—represent the most realistic pathway toward mass-produced, chloride sensors. These approaches balance performance, cost, and scalability, aligning materials design with real-world constraints rather than idealized laboratory conditions.

## 3. Theoretical foundations and governing equations of halide exchange in lead halide perovskites

### 3.1. Crystal structure and intrinsic ionic conductivity

CsPbX_3_ perovskites belong to the ABX_3_ family and exhibit borderline structural stability governed by the Goldschmidt tolerance factor (*t* ≈ 0.92 for CsPbBr_3_; *t* ≈ 0.85 for CsPbCl_3_). This proximity to the cubic–orthorhombic phase boundary results in a structurally soft lattice with low-energy phonon modes and low halide vacancy migration barriers (VBr^+^ ≈ 0.25–0.30 eV; VCl^+^ ≈ 0.35 eV).^58,59^ Consequently, intrinsic anionic conductivity (10^−8^–10^−6^ S cm^−1^ at 300 K) is several orders of magnitude higher than in conventional quantum dots, enabling rapid halide exchange on a seconds timescale.

**Fig. 1:**
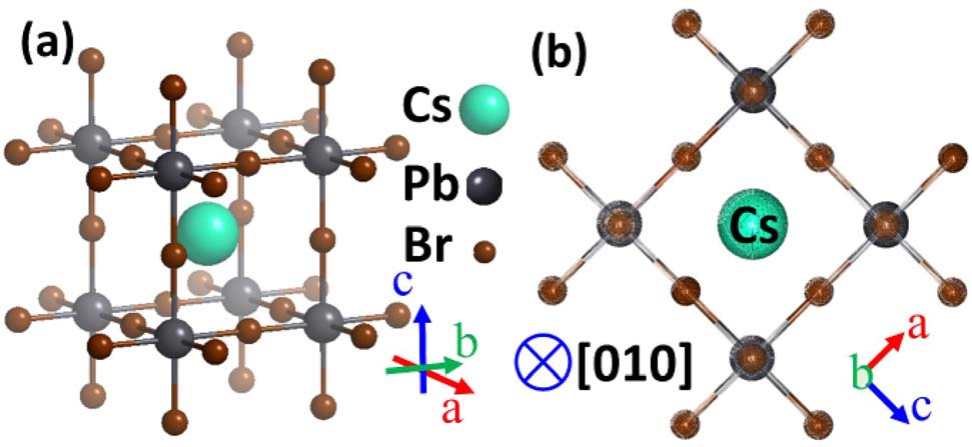
(a) Cubic *Pm*3̄*m* crystal structure of CsPbBr_3_ showing the ABX_3_ perovskite framework. (b) [010] projection highlighting continuous Pb–Br connectivity and intrinsic halide diffusion pathways relevant to fast Br-/Cl-exchange. Adapted with permission from ref. 80. © 2025 American Chemical Society.

Fig. 1 illustrates the cubic *Pm*3̄*m* structure of CsPbBr_3_ and its projection along [010], highlighting the continuous Pb–Br framework and diffusion pathways responsible for vacancy-assisted halide migration. The preservation of the cationic sublattice during early-stage exchange explains the initial retention of the cubic phase prior to gradual mixed-halide phase evolution.^80^

### 3.2. Lattice parameter evolution (Vegard's law)

The CsPb(Br_1−*x*_Cl_*x*_)_3_ system forms a continuous solid solution obeying Vegard's law within 0.2% deviation:^60,61^

1 *a*(*x*) = 5.874 − 0.269*x* Å

The linear lattice contraction provides a calibration-free structural method to quantify chloride incorporation.

**Fig. 2:**
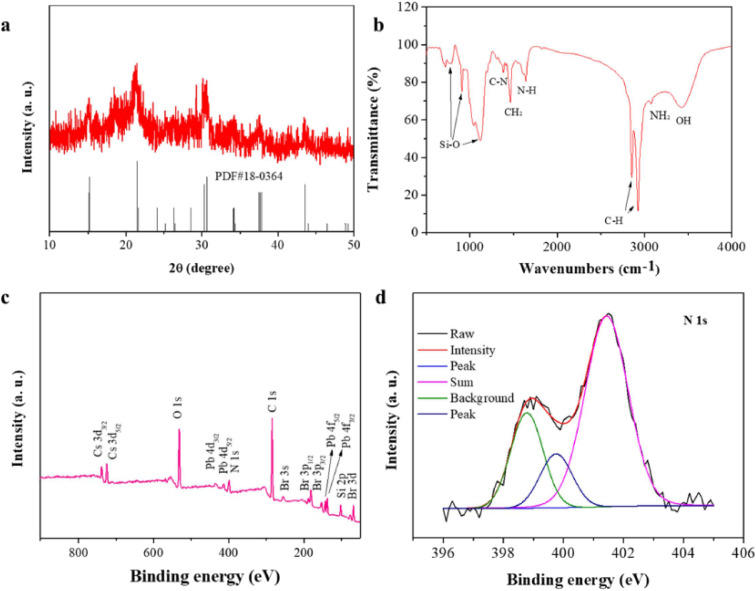
Structural and interfacial characterization of CsPbBr_3_@SiO_2_ nanocrystal composites. (a) X-ray diffraction pattern confirming the preservation of the cubic CsPbBr_3_ crystal structure after incorporation into the SiO_2_ matrix. (b) FT-IR spectrum showing characteristic vibrational features associated with the silica network surrounding the nanocrystals. (c) XPS survey spectrum of the composite material. (d) High-resolution N 1s spectrum indicating the presence of nitrogen-containing surface functionalities that contribute to interfacial stabilization and surface passivation. Adapted with permission from ref. 87. © 2022 MDPI.

At this point, it is important to clarify the applicability of these optoelectronic models in a sensing context. While lattice contraction, bandgap bowing, and Vegard-type relationships are conventionally employed to describe steady-state behavior in LEDs and photodetectors, halide-exchange-based sensing operates under fundamentally different conditions. In sensing, the optical response reflects a dynamic, kinetically controlled process in which ion diffusion, surface reaction rates, and interfacial energetics govern time-dependent changes in composition and band structure. Nevertheless, the underlying structure–property relationships remain valid because the perovskite framework retains its optoelectronic integrity during exchange. This continuity is experimentally supported by the preserved crystallinity and interfacial stabilization observed in Fig. 2, which indicate that halide substitution proceeds without irreversible lattice collapse. Accordingly, steady-state optoelectronic models can be extended to sensing applications by incorporating dynamic descriptors such as diffusion coefficients and exchange rate constants.

### 3.3. Bandgap and optical bowing

The bandgap follows a quadratic bowing relation:^62,63^

2 *E*_g(*x*)_ = 2.35(1 − *x*) + 3.12*x* − *b x*(1 − *x*), *b* ≈ 1.0 eV

The positive bowing arises from orbital mismatch, lattice deformation, and strong ionic character. This relation underpins wavelength-shift-based chloride sensing.

### 3.4. Photoluminescence–composition correlation

The empirical PL relation:^64,65^

3 *λ*_PL_ = 522.4 − 112.8*x* + 29.6*x*^2^

combined with

4 *E*_g_ = 1239.8/*λ*_PL_

Empirical correlations between photoluminescence (PL) wavelength and halide composition are widely used to quantify chloride incorporation in CsPb(Br_1−*x*_Cl_*x*_)_3_ nanocrystals. The quadratic PL–composition relation (eqn (3)), combined with the photon energy conversion (eqn (4)), provides a practical framework for wavelength-based chloride sensing. However, recent computational and *operando* studies indicate that this relationship should be regarded as an effective, phenomenological descriptor rather than a strict equilibrium mapping, particularly at the nanoscale.

Density functional theory calculations reveal that halide exchange is governed by vacancy-assisted migration within a structurally soft Pb–X lattice, with low activation barriers and strong coupling between ionic motion and lattice deformation. Importantly, these studies show that band-edge energies respond to local halide configurations and transient lattice distortions rather than to average composition alone. Molecular dynamics simulations further demonstrate significant dynamic disorder, where halide ions undergo continuous thermal fluctuations, producing time-averaged bandgaps that depend on temperature, surface termination, and ligand interactions.

*Operando* spectroscopic and diffraction studies provide experimental validation of these predictions. Time-resolved PL measurements during halide exchange often show continuous wavelength shifts preceding full lattice equilibration, indicating that optical response is dominated by early-stage surface substitution and near-surface halide gradients. In several systems, PL evolution occurs without immediate changes in long-range crystal structure, confirming that emission wavelength probes local electronic environments rather than bulk compositional homogeneity. Under vapor-phase or high-flux conditions, *operando* studies further reveal that lattice dynamics and transient phase evolution can enhance sensitivity while introducing hysteresis and environmental dependence. Together, these insights refine the interpretation of PL–composition correlations, highlighting that wavelength-based calibration averages over dynamic, non-equilibrium halide distributions while remaining highly effective for practical sensing applications.

### 3.5. Exchange kinetics

Halide exchange follows pseudo-first-order kinetics:^66,67^

5 ln[(*λ*_∞_ − *λ*_*t*_)/(*λ*_∞_ − *λ*_0_)] = −*k*_obs_*t*

with activation energies of 22–40 kJ mol^−1^, explaining diffusion-limited yet ultrafast exchange under ambient conditions.

### 3.6. Thermodynamic equilibrium

The exchange equilibrium:^68,69^

6 Br^−^ + Cl^−^ ⇌ Cl^−^ + Br^−^

7 *K*_ex_ ≈ 0.08 − 0.30

favors partial chloride incorporation, ensuring reversibility and preventing irreversible lattice collapse.

### 3.7. Exciton binding and optical robustness

Exciton binding energy remains 8–15 meV even at high chloride fractions,^70,71^ maintaining high PLQY (>80%). Weak quantum confinement adds size-dependent shifts without disrupting linear wavelength–composition calibration.^72–74^ These validated structural, thermodynamic, kinetic, and optical relationships collectively define the mechanistic basis of halide-exchange-driven optical transduction in CsPbBr_3_ nanocrystals.^75,76^

### 3.8. Limitations of Vegard's law and bandgap bowing models in nanocrystalline CsPb(Br_1−*x*_Cl_*x*_)_3_ systems

Vegard's law and quadratic bandgap bowing models are widely employed to correlate lattice parameters, bandgap energy, and photoluminescence wavelength with halide composition in mixed-halide perovskites. While these relationships provide an effective first-order approximation in bulk and thin-film CsPb(Br_1−*x*_Cl_*x*_)_3_, their direct application to nanocrystalline systems requires careful consideration. At the nanoscale, several physical effects introduce systematic deviations that limit the quantitative accuracy of these models if applied without correction.

One major limitation arises from quantum confinement effects. In CsPbBr_3_ nanocrystals with sizes comparable to or smaller than the exciton Bohr radius, bandgap energies are shifted relative to bulk values even at fixed composition.^47,49^ As a result, identical chloride fractions may yield different emission wavelengths depending on nanocrystal size. Although confinement-induced shifts are often approximately additive to compositional bandgap changes, this assumption breaks down in strongly confined or polydisperse systems, leading to non-unique wavelength–composition mappings.

A second source of deviation is surface-induced lattice strain. Nanocrystals exhibit a high surface-to-volume ratio, and surface ligands, encapsulation layers, or interfacial bonding to matrices (*e.g.*, silica or polymers) can impose compressive or tensile strain on the perovskite lattice. Such strain modifies lattice parameters independently of composition, causing departures from linear Vegard behavior. Consequently, XRD-derived lattice constants may overestimate or underestimate actual chloride incorporation if surface strain is not accounted for.

Compositional heterogeneity further limits the applicability of bulk models. Halide exchange in nanocrystals is often surface-initiated and diffusion-limited, producing transient or persistent core–shell-like halide gradients rather than homogeneous solid solutions. In these cases, the measured optical response represents an average over non-uniform compositions, whereas Vegard's law and bowing equations implicitly assume a spatially uniform lattice.^51,53^ This effect becomes particularly pronounced at short exchange times or in partially encapsulated systems where ion diffusion is restricted.

Additionally, non-equilibrium and kinetic effects challenge the thermodynamic assumptions underlying these models. Vegard's law and bowing relations describe equilibrium solid solutions, whereas halide exchange during sensing frequently operates under dynamic, non-equilibrium conditions. In such regimes, emission wavelength may reflect kinetically trapped states rather than equilibrium compositions, leading to hysteresis or time-dependent calibration shifts. These limitations do not invalidate the use of Vegard's law or bowing models in nanocrystal-based chloride sensing but instead define their domain of reliability. In practice, accurate quantification at the nanoscale requires size-specific calibration, complementary structural characterization, or empirical correction factors tailored to a given nanocrystal architecture. Recognizing these constraints is essential for transitioning halide-exchange sensing from qualitative or semi-quantitative demonstrations toward predictive and standardized analytical platforms.

### 3.9. Position of CsPbBr_3_ within the broader halide-perovskite sensing landscape

Although this review centers on CsPbBr_3_ nanocrystals as a representative platform for chloride sensing, it is useful to consider how the mechanistic concepts discussed here relate to the broader family of halide perovskites. Across the CsPbX_3_ series (X = Cl, Br, I), halide exchange is generally governed by similar physicochemical factors, including lattice flexibility, vacancy-assisted ion migration, surface accessibility, and the dynamic equilibrium of surface ligands. These shared features make halide perovskites particularly attractive for sensing applications in which compositional changes can be directly translated into optical signals such as absorption or photoluminescence shifts.

Within this family, CsPbBr_3_ occupies a particularly advantageous position for chloride sensing studies. Compared with iodide-rich compositions such as CsPbI_3_, CsPbBr_3_ typically exhibits improved phase stability and greater tolerance to environmental perturbations, while still maintaining sufficiently soft lattice characteristics that enable rapid halide exchange.^35–37^ At the same time, the substitution of Br^−^ by Cl^−^ produces a pronounced blue shift in the optical spectra, generating a clear and quantifiable signal that is well suited for colorimetric and fluorimetric detection strategies.

Chloride-dominant systems such as CsPbCl_3_, by contrast, may exhibit a narrower compositional window for chloride incorporation, which can reduce the magnitude of detectable spectral shifts in chloride sensing scenarios. Conversely, iodide-rich materials often display enhanced ionic mobility due to increased lattice softness, but they may suffer from reduced environmental and phase stability, which complicates their practical use in aqueous or ambient sensing environments.

Hybrid and quasi-two-dimensional perovskite structures introduce additional design possibilities through the incorporation of organic spacer layers or interfacial barriers. These architectures can enhance environmental robustness and introduce selective ion-transport pathways, potentially improving durability or selectivity in sensing applications.^22–24^ However, the presence of organic layers can also alter diffusion pathways and exchange kinetics, making their sensing response more dependent on interfacial transport processes.

Taken together, these comparisons indicate that while CsPbBr_3_ provides a convenient and well-characterized model system for studying halide-exchange-based sensing, the underlying structure–exchange–stability–response relationships discussed in this review are broadly applicable across halide perovskite materials. Nevertheless, the practical implementation of these concepts must account for composition-dependent differences in lattice stability, ion transport, and optical response.

## 4. Mechanistic insights and functional dynamics of CsPbBr_3_ nanocrystals for chloride ion sensing

### 4.1. Comparative mechanistic pathways of halide exchange across media

Halide exchange in CsPbBr_3_ nanocrystals operates through a common vacancy-assisted substitution mechanism, yet comparative analysis across liquid, biphasic, and vapor systems reveals distinct kinetic and structural regimes rather than a single universal behavior. In pure aqueous systems using water-stable W-PNCs, exchange proceeds directly at the nanocrystal surface without encapsulation barriers, enabling fast diffusion-controlled substitution and enabling smartphone-integrated detection platforms.^79^ In contrast, biphasic systems (*e.g.*, *n*-hexane/aqueous sweat detection) rely on interfacial ion transport under acidic conditions (pH ≈ 1), where protonation of ligands enhances chloride permeability and accelerates exchange.^78^ Vapor-phase detection of HCl or chlorinated volatiles introduces an additional mechanistic layer: exchange is coupled to solid-state phase transitions and lattice rearrangement, as confirmed by XRD-resolved cubic-to-tetragonal-to-orthorhombic evolution.^80,86^

Cross-comparison shows that response time is governed less by intrinsic lattice mobility (already high) and more by interfacial accessibility. Systems without compact coatings exhibit sub-second to few-second responses,^78,84^ whereas coated composites such as SiO_2_-modified nanocrystals show slightly slower but more controlled kinetics.^87^ Importantly, reversible exchange dominates in aqueous systems,^79,84^ while vapor-phase detection may involve deeper structural reorganization that enhances sensitivity but increases dependence on environmental conditions.^80^

Another key trend concerns halide identity. Comparative Cl^−^/I^−^ studies demonstrate asymmetric exchange behavior: chloride induces predictable blue shifts with modest lattice strain, while iodide substitution introduces larger steric perturbation and slower equilibration.^81,90^ This comparative evidence indicates that selectivity in CsPbBr_3_ sensing arises not merely from ion recognition but from differential kinetic accessibility and lattice strain accommodation. Overall, synthesis of these studies suggests that halide exchange is best described as a surface-governed, medium-dependent dynamic equilibrium process whose analytical performance depends primarily on diffusion pathways and interfacial design rather than bulk lattice limitations.

Fig. 2 integrates conceptual modeling and time-resolved photoluminescence evidence for halide exchange in water-dispersed CsPbBr_3_ systems. Panels (a and b) illustrate surface-initiated Br^−^/Cl^−^ substitution and inward diffusion without structural collapse.^79^ Panels (c–e) show continuous wavelength evolution during Cl^−^ exchange, highlighting two kinetic regimes (rapid surface substitution followed by slower bulk equilibration). Panels (f–h) compare iodide exchange dynamics, demonstrating halide-dependent spectral trajectories and validating kinetic selectivity mechanisms.

It is important to emphasize that photoluminescence wavelength shifts do not, by themselves, uniquely prove stoichiometric halide exchange. Alternative mechanisms such as surface reconstruction, partial lattice degradation, or nanoscale phase segregation can also modify emission energies. Surface reconstruction, driven by ligand reorganization or defect redistribution, may induce modest blue shifts without bulk halide incorporation, typically accompanied by PL intensity loss or spectral broadening. In contrast, degradation processes—such as PbBr_2_ formation or hydration-induced amorphization—often produce irreversible quenching and discontinuous spectral changes rather than smooth, composition-dependent shifts.

Phase segregation represents another potential confounding factor, particularly at high halide flux or under illumination. In such cases, coexistence of Br-rich and Cl-rich domains may yield apparent intermediate emission wavelengths arising from ensemble averaging rather than homogeneous solid solutions. Distinguishing true halide exchange from these effects requires converging evidence: continuous and reversible wavelength evolution, retention of perovskite diffraction signatures, and regeneration of the original emission upon bromide reintroduction. Studies satisfying these criteria consistently support vacancy-assisted halide substitution as the dominant mechanism, whereas systems lacking reversibility or structural retention likely involve competing surface or degradation-driven processes.

### 4.2. Reversibility and phase stability: reconciling conflicting reports

A careful examination of the structural and interfacial characteristics of halide-perovskite nanocrystals is essential for understanding why reports of reversibility and phase stability sometimes appear inconsistent across the literature. In many sensing environments, particularly aqueous or biphasic systems, halide exchange is largely governed by surface processes rather than bulk lattice transformation. Under such conditions, structural retention of the perovskite framework becomes a critical factor that determines whether optical responses can be regenerated after halide re-equilibration. Fig. 2 therefore provides relevant structural and spectroscopic evidence that helps contextualize how interfacial stabilization and lattice preservation contribute to reversible behavior in CsPbBr_3_-based systems.

The X-ray diffraction (XRD) patterns shown in Fig. 2a confirm the preservation of the crystalline CsPbBr_3_ phase after encapsulation within the SiO_2_ matrix. The characteristic diffraction peaks associated with the cubic perovskite lattice remain clearly identifiable, indicating that the structural framework of the nanocrystals is maintained during the formation of the composite architecture. Such structural retention is particularly relevant for sensing scenarios where repeated ion-exchange cycles occur, because the ability of the lattice to maintain its crystallographic integrity is a prerequisite for reversible optical responses. In this context, the diffraction data support the notion that the perovskite core can withstand interfacial exchange processes without undergoing irreversible phase degradation.

Complementary spectroscopic information provided by the FT-IR spectra in Fig. 2b offers insight into the chemical environment surrounding the nanocrystals. The presence of vibrational features associated with Si–O–Si linkages confirms the formation of a crosslinked silica network that surrounds or anchors the CsPbBr_3_ nanocrystals. Such inorganic encapsulation can act as a stabilizing framework that moderates the accessibility of halide ions to the perovskite lattice while simultaneously protecting the nanocrystals from environmental degradation. In sensing contexts, this type of interfacial architecture can favor surface-confined exchange processes, thereby reducing the likelihood of deep lattice reconstruction that could compromise reversibility.

Further evidence for interfacial stabilization is provided by the XPS spectra and the high-resolution N 1s signal presented in Fig. 2c and d. These spectra indicate the presence of nitrogen-containing surface functionalities that can interact with the perovskite surface and contribute to defect passivation or anchoring within the surrounding matrix. Such surface interactions are known to suppress non-radiative recombination pathways and improve the chemical robustness of perovskite nanocrystals. Importantly, controlled surface passivation may also regulate ion migration pathways, thereby supporting exchange processes that remain largely confined to the near-surface region. This behavior is consistent with the mechanistic interpretation proposed in Section 4.1.1, where differences in reversibility across studies are attributed primarily to variations in exchange depth and environmental conditions rather than to fundamentally contradictory material behavior.

### 4.3. Optical transduction: sensitivity, linearity, and phase-dependent behavior

Across reported systems, optical transduction consistently manifests as a blue shift in absorption and photoluminescence spectra proportional to chloride incorporation; however, quantitative performance varies significantly depending on matrix, architecture, and stabilization strategy. Direct aqueous sensing platforms demonstrate linear detection ranges spanning micromolar to millimolar concentrations, with LODs as low as 4 µM in domestic water^89^ and 3.2 × 10^−6^ M in engineered hydrogen-bonded systems.^81^ In urine and biosensing contexts, linearity typically extends from 2–200 mM with rapid response (∼1 s), prioritizing clinical practicality over ultralow detection limits.^84^

Comparatively, vapor-phase detection achieves exceptional sensitivity (down to 0.005 ppm for HCl),^80^ but this high sensitivity is coupled to phase-transition-driven lattice evolution, which introduces dependence on environmental stability and irradiation conditions. Photochromic detection of dichloromethane illustrates another transduction mode, where UV-catalyzed chloride release leads to wavelength shifts correlated with solvent concentration.^85^ These examples collectively demonstrate that while wavelength shift remains the universal signal, the underlying transduction pathway (direct ion exchange *vs.* photochemical generation *vs.* phase evolution) influences calibration behavior and reproducibility.

Encapsulation strategies introduce an additional trade-off. SiO_2_-coated composites preserve linear wavelength shifts in semi-aqueous matrices,^87^ but diffusion barriers slightly reduce kinetic response compared to uncoated water-dispersed PNCs.^79^ Ligand-engineered hydrophilic systems balance permeability and stability, achieving reproducible detection in biological fluids without sample pretreatment.^84,88^ Critically, cross-study comparison indicates that wavelength-based sensing is inherently more than intensity-based approaches because it decouples analytical signal from absolute PLQY fluctuations. However, spectral broadening at high chloride concentration and matrix-dependent strain effects necessitate system-specific calibration, particularly in confined nanocrystals where size-dependent bandgap shifts may overlap with compositional effects.^81,82^

**Fig. 3:**
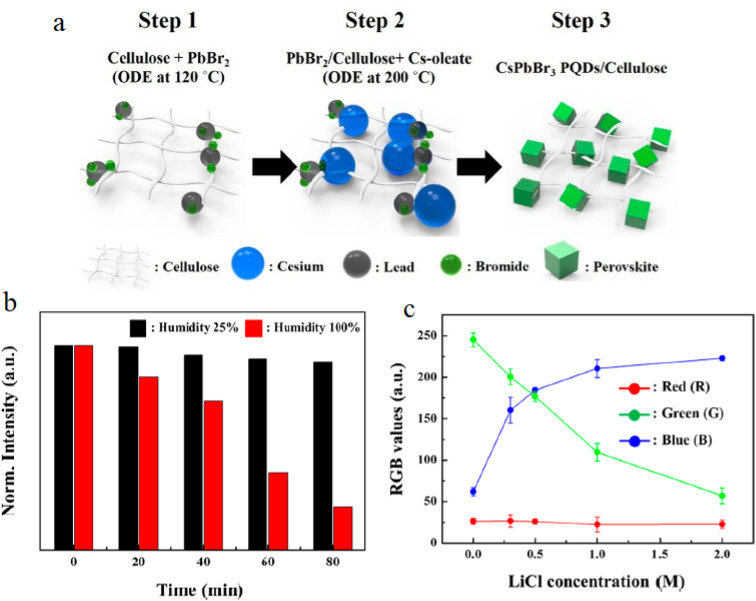
(a) Stepwise nucleation and growth of CsPbBr_3_ PQDs on cellulose nanofibers. (b) Photoluminescence stability under ambient and high-humidity conditions. (c) RGB color response as a function of chloride concentration, demonstrating halide-exchange-based sensing. Adapted with permission from ref. 83. © 2019 American Chemical Society.

Thus, the dominant trend across studies is not merely high sensitivity, but the need to optimize the balance between kinetic accessibility, spectral linearity, and environmental. Fig. 3 demonstrates matrix-assisted stabilization and colorimetric transduction in CsPbBr_3_ PQDs/cellulose composites.^83^ Panel (a) illustrates controlled nucleation of PQDs on cellulose fibers, limiting aggregation. Panel (b) compares photoluminescence stability under varying humidity, confirming structural protection. Panel (c) shows RGB-based color evolution as a function of chloride concentration, validating practical semi-quantitative detection without instrumentation.

### 4.4. Stability engineering *versus* exchange kinetics: trade-offs and design principles

A consistent cross-study trend is the inherent tension between structural stability and ion-exchange accessibility. Uncoated or minimally protected water-dispersed nanocrystals exhibit superior sensitivity and rapid exchange kinetics,^79^ yet suffer from limited long-term environmental durability. Conversely, encapsulated systems such as CsPbBr_3_@SiO_2_ composites dramatically improve resistance to ethanol and water exposure,^87^ but introduce diffusion barriers that modestly slow halide exchange. Ligand engineering provides an intermediate strategy. Hydrogen-bonded β-cyclodextrin–arginine networks allow selective halide permeation while stabilizing the lattice against humidity and thermal stress.^81^ Amphiphilic polymer ligands reduce surface trap density and preserve up to 80% photoluminescence after prolonged aqueous exposure,^88^ while maintaining sufficient accessibility for chloride detection in sweat matrices. Hydrophilic micellar glycyrrhizic acid coatings enable ethanol dispersion and wide linear detection ranges in urine sensing.^84^

Comparative analysis reveals that optimal sensor performance does not arise from maximum encapsulation, but from controlled permeability that preserves reversible exchange while suppressing degradation. Stability enhancements are particularly critical in industrial wastewater monitoring devices, where long-term operational accuracy (∼98.9%) has been demonstrated only when interfacial and reproducibility are engineered simultaneously.^77^ Phase-transition studies further highlight this balance: while structural evolution during exchange can enhance sensitivity in vapor detection,^80^ uncontrolled transitions under humidity may reduce reproducibility.^83^ Therefore, design principles emerging from these studies emphasize moderated ligand binding strength, diffusion-channel engineering, and defect passivation without complete surface isolation.

**Fig. 4:**
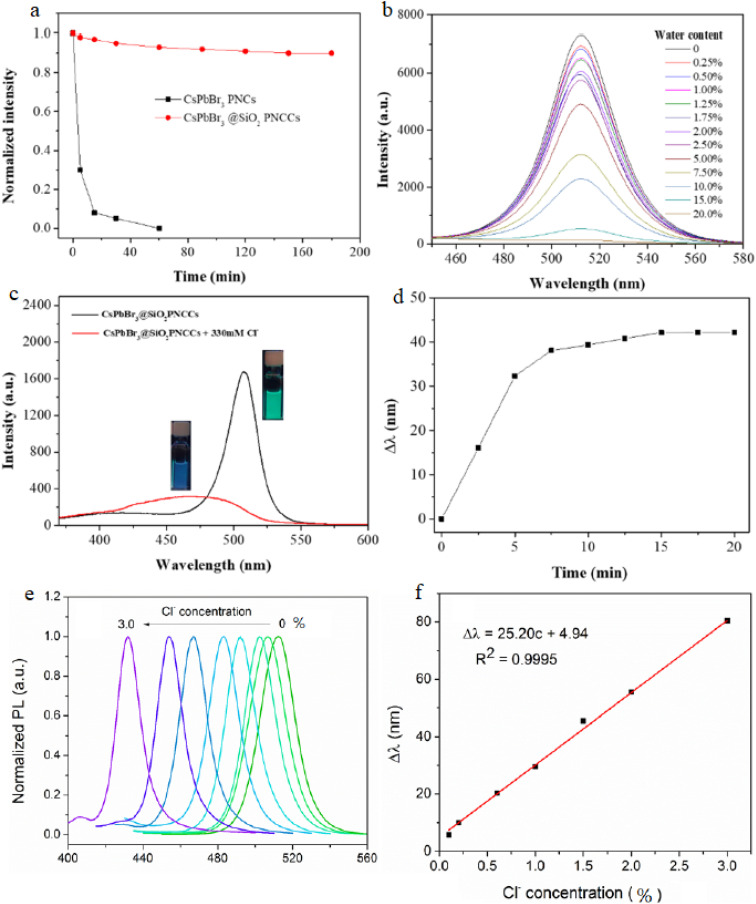
(a) Fluorescence stability of bare CsPbBr_3_ nanocrystals and CsPbBr_3_@SiO_2_ composites in ethanol. (b) Effect of water content on photoluminescence stability. (c and d) Homogeneous halide exchange with Cl^−^ ions. (e and f) Emission wavelength shift and calibration curve for chloride sensing under UV excitation (365 nm). Adapted with permission from ref. 87. © 2022 MDPI.

In synthesis, the field is converging toward hybrid stabilization strategies that balance kinetic responsiveness and environmental durability rather than prioritizing one at the expense of the other. Fig. 4 compares stability and sensing performance of bare CsPbBr_3_ and SiO_2_-coated composites.^87^ Panel (a) shows rapid fluorescence decay of uncoated nanocrystals in ethanol *versus* preserved emission in coated systems. Panel (b) illustrates gradual PL attenuation with increasing water content. Panels (c–f) demonstrate homogeneous halide exchange, emission blue shift, and linear calibration behavior in semi-aqueous matrices, confirming improved without complete loss of sensitivity.

### 4.5. Multi-matrix applicability: from biological fluids to industrial effluents

A central question in evaluating CsPbBr_3_-based chloride sensors is whether their performance is intrinsically limited to ideal laboratory media or whether it remains reliable across chemically complex real-world matrices. Comparative analysis of recent reports demonstrates that the halide-exchange mechanism is fundamentall, while analytical performance depends primarily on interfacial compatibility with the surrounding environment. In relatively clean aqueous systems such as drinking water, detection limits can reach the micromolar range (LOD ≈ 4 µM) with linear calibration behavior, reflecting minimal interference and efficient ion accessibility.^89^ Under these conditions, surface-exposed nanocrystals provide rapid equilibrium and high signal fidelity.

In contrast, biological fluids such as sweat and urine introduce high ionic strength, organic components, and competing ions that may perturb ligand shells or induce nonspecific quenching. Studies employing hydrophilic ligand engineering or micellar encapsulation demonstrate that maintaining controlled surface permeability is sufficient to preserve rapid response (∼1 s) and wide linear detection windows (2–200 mM) even in physiologically relevant media.^84,88^ This indicates that matrix complexity does not fundamentally suppress halide exchange but instead challenges surface stabilization strategies.

Industrial wastewater presents an even harsher environment, often characterized by fluctuating pH, mixed ionic species, and potential heavy-metal contaminants. Devices optimized for these conditions emphasize reproducibility and long-term operational accuracy rather than absolute sensitivity, achieving analytical reliability approaching 98.9% under field conditions.^77^ The comparative trend across matrices reveals that while detection limits vary, the core wavelength-shift transduction remains stable provided that surface degradation is mitigated. Taken together, these studies demonstrate that CsPbBr_3_ sensing platforms are highly adaptable. Performance differences arise not from changes in intrinsic lattice exchange thermodynamics, but from matrix-induced modifications of diffusion pathways and surface chemistry. Thus, matrix adaptability emerges as a function of ligand selection and interfacial design rather than a limitation of the perovskite core itself.

### 4.6. Selectivity and competitive ion effects

Selectivity in halide-exchange-based sensing differs fundamentally from receptor-based molecular recognition. Rather than relying on specific binding motifs, CsPbBr_3_ nanocrystals discriminate ions through lattice substitution energetics and kinetic accessibility. Comparative studies consistently show that non-halide anions such as NO_3_^−^, SO_4_^2−^, and PO_4_^3−^ do not induce measurable bandgap shifts, as they cannot be incorporated into the perovskite lattice.^81,89^ Consequently, wavelength-based sensing is intrinsically resistant to interference from structurally incompatible species. Within the halide family, however, substitution behavior differs markedly. Chloride incorporation produces predictable blue shifts with modest lattice contraction and rapid equilibration, whereas iodide substitution induces lattice expansion and comparatively slower exchange kinetics.^81,90^ This asymmetry originates from ionic radius mismatch and strain accommodation within the Pb–X framework. As a result, Cl^−^ detection benefits from both kinetic favorability and structural compatibility, enhancing analytical reliability.

Surface engineering further modulates selectivity. Hydrogen-bonded supramolecular coatings and amphiphilic ligands can enrich chloride concentration near the nanocrystal interface without permanently blocking diffusion channels.^81,88^ Such strategies increase effective local ion activity and improve signal-to-noise ratio under competitive conditions. However, excessive encapsulation may suppress ion accessibility, illustrating again the trade-off between stability and responsiveness. Importantly, comparative evidence suggests that spectral peak position is a more selective metric than intensity changes. Competing ions may influence surface trap states and alter photoluminescence intensity, but only halide substitution directly modifies the band structure and emission wavelength. Therefore, selectivity is best conceptualized as structurally encoded discrimination embedded in the lattice itself rather than externally imposed chemical recognition.

### 4.7. Reversibility, regeneration, and operational lifetime

A defining advantage of CsPbBr_3_-based chloride sensing is the reversibility of halide exchange. Unlike irreversible colorimetric reactions, chloride incorporation into the lattice represents a near-equilibrium substitution process that can be reversed through bromide reintroduction.^79^ This reversibility enables cyclic operation, an essential feature for reusable analytical devices. Comparative studies reveal that regeneration efficiency depends strongly on surface stabilization. Bare nanocrystals exhibit ultrafast exchange kinetics but may accumulate surface defects over repeated cycles, gradually reducing photoluminescence intensity. In contrast, polymer- and silica-stabilized systems maintain structural integrity across multiple exchange–regeneration cycles, preserving spectral reproducibility even under semi-aqueous conditions.^87,88^ This suggests that long-term durability is primarily governed by defect management rather than by intrinsic thermodynamic limitations.

Vapor-phase sensing presents a distinct case. Structural phase transitions accompanying chloride uptake can amplify sensitivity but may also introduce partial irreversibility under uncontrolled humidity or prolonged exposure.^80^ Thus, operational lifetime in gas-sensing configurations is influenced by environmental regulation as much as by lattice chemistry. Field-deployed wastewater monitoring devices demonstrate that sustained analytical accuracy (∼98.9%) is achievable when stabilization strategies balance diffusion accessibility with structural protection.^77^ The emerging consensus is that optimal operational lifetime is obtained not by suppressing exchange, but by ensuring that reversible ion diffusion proceeds without cumulative surface degradation. Consequently, regeneration capability must be integrated into material design rather than considered a secondary attribute.

### 4.8. Emerging design principles and future integration pathways

When the collective body of work is examined comparatively, several coherent design principles emerge. First, rapid halide exchange is inherently supported by high anionic conductivity; therefore, performance optimization should focus on surface engineering rather than bulk structural modification. Second, wavelength shift constitutes a structurally encoded analytical signal that is inherently more against environmental fluctuations than intensity-based metrics. Third, moderate encapsulation strategies that allow controlled permeability consistently outperform either fully exposed or fully isolated architectures.

Integration into practical platforms further illustrates the maturity of this sensing concept. Smartphone-assisted RGB quantification enables instrument-free semi-quantitative analysis in composite films,^83^ while portable wastewater monitoring systems demonstrate real-time applicability in industrial contexts.^77^ Wearable-compatible matrices and hydrophilic ligand systems extend applicability to biomedical monitoring scenarios.^84^ These examples confirm that halide-exchange nanocrystals can bridge nanoscale photophysics and macroscale analytical deployment.

Future development pathways include multiplexed halide discrimination, microfluidic integration for automated sampling, and algorithm-assisted spectral deconvolution to improve quantification accuracy in complex matrices. Additionally, exploration of lead-reduced compositions may address environmental considerations while retaining exchange-driven optical tunability. Overall, the field is transitioning from proof-of-concept demonstrations toward platform-level optimization grounded in predictable lattice chemistry, reversible ion substitution, and engineered interfacial control. This convergence of structural understanding and practical deployment underscores the technological relevance of CsPbBr_3_-based halide-exchange sensing systems.

Table 2 compiles key performance indicators from the referenced studies, highlighting variations in detection limits, response speeds, and practical utilities across different media and formats. It reveals a trend toward lower LODs in advanced ligand-engineered systems (*e.g.*, micromolar to nanomolar ranges), while emphasizing the trade-offs between rapidity and specificity in complex environments. Notably, vapor and photo-assisted methods extend beyond liquid phases, achieving sub-ppm sensitivities, whereas portable integrations prioritize user accessibility without sacrificing reliability, underscoring the adaptability of CsPbBr_3_ PNCs for diverse analytical demands.

**Table 2:** Quantitative performance benchmarks of CsPbBr_3_-based chloride sensors

Detection strategy	LOD	Linear range	Response time	Sample/application	Stability & robustness	Ref.
β-CD/arginine-capped CsPbBr_3_ PNCs (H-bond cross-linked)	3.2 µM	5–200 µM	<10 s	Cl^−^/I^−^ in water, sweat, saliva	PLQY ≈ 82%; stable under heat & humidity; RSD ≈ 5.7%	81
Gas-phase halide exchange (HCl vapor)	5 ppm	5–100 ppm	∼3 s	Toxic HCl gas	Crystal morphology preserved; reversible PL shift	86
Solution/film/paper-strip halide exchange	100 µM	0.1–5 mM	<1 s (instantaneous)	On-site water detection	Stable PL under ambient conditions; visual read-out	82
Biphasic halide exchange (*n*-hexane/aqueous)	≈1 mM (estimated)	0.2–10 mM (typical for sweat range)	∼5 min	Sweat Cl^−^ (cystic fibrosis)	No pretreatment; works at pH 1; strong color contrast	78
CPB-FC-Cl portable device	≈50 µM (estimated)	0–1 g L^−1^	∼5 min	Industrial wastewater	Accuracy ≈ 99%; multi-cycle stability ≈ 99%; durable electronics	77
GA-capped hydrophilic PNCs	1.82 mM	2–200 mM	∼1 s	Urine chloride	PLQY≈59%; robust across pH 2–10	84
Water-dispersible W–PNCs	≈10 µM (estimated)	10–500 µM	∼10 s	Halides in pure water	High fluorescence; direct aqueous sensing	79
CsPbBr_3_@SiO_2_ nanocomposites	≈0.5 wt% (≈5 mM)	0–3.0 wt%	<1 min	Cl^−^ in sea-sand samples	Improved moisture stability; uniform exchange	87
PQDs/cellulose composite	≈10 µM (estimated)	10–200 µM	<5 s	Tap-water Cl^−^/I^−^	Resistant to humidity, pH, temperature	83
Wavelength-shift colorimetric sensing (solution)	4 µM	10–200 µM	<3 s (photo-response)	Domestic water Cl^−^	PLQY≈87%; recovery 99–104%	89
OPA-capped CsPbBr_3_ NCs	≈ 20 µM (estimated)	5–100 µM	∼2 s	Sweat chloride	80% fluorescence retained after 15 days; thermally stable	88
Thin-film PQDs (phase-transition sensing)	0.005 ppm (HCl)	0.005–10 ppm	∼5 s	HCl/NaOCl vapors	UV-assisted LOD 0.50 ppm (NaOCl); sequential phase changes	80
UV-photochromic halide sensing	0.29% DCM	1–20%	30 min (under UV)	Environmental DCM	Reversible PL color shift; stable to multiple UV cycles	85
Spectrochemical halide-exchange catalyst	≈50 µM (estimated)	50–500 µM	Real-time (<1 s)	Reaction monitoring	Acts as halide reservoir; fast colorimetric kinetics	90

## 5. Limitations, challenges, and industrial prospects of CsPbBr_3_-based chloride sensors

### 5.1. Environmental and chemical stability constraints

CsPbBr_3_ PNCs have demonstrated exceptional PL and halide exchange responsiveness, yet their practical deployment is often constrained by inherent environmental and chemical instabilities.^81^ Exposure to moisture, oxygen, polar solvents, and variable pH conditions can induce structural degradation, resulting in diminished fluorescence efficiency and compromised sensing performance. While encapsulation strategies—such as polymer or inorganic coatings—can mitigate these effects, they frequently restrict direct ion accessibility, thereby reducing the sensitivity and response speed of the halide exchange process.^79,81^

Another critical limitation stems from thermal instability, as CsPbBr_3_ PNCs are prone to lattice deformation at elevated temperatures, leading to phase transitions that irreversibly alter photophysical properties.^80^ This thermal sensitivity not only limits applications in industrial or environmental monitoring but also poses challenges for storage and transport of sensor materials. Hydrogen-bonding networks or ligand engineering (*e.g.*, β-cyclodextrin-arginine or amphiphilic polymer capping) have been proposed,^88^ yet scalability and reproducibility remain challenging.

Chemical stability is further complicated by the dynamic nature of halide exchange. While rapid exchange kinetics enables fast detection, they also make PNCs susceptible to unintended interactions with ambient ions or contaminants. For instance, coexisting halides or organohalides in complex matrices may induce partial or non-stoichiometric exchanges, yielding ambiguous or less reproducible fluorescence shifts.^90^ This emphasizes the need for careful matrix-specific calibration when deploying these nanocrystals for real-world monitoring.

Finally, photostability under prolonged illumination is a concern for continuous or long-term sensing applications. Photo-induced degradation or ion migration within the perovskite lattice can gradually decrease emission intensity, limiting operational lifetime.^88^ Despite progress with ligand-assisted stabilization and encapsulation, further research is required to engineer intrinsically stable PNCs capable of retaining optical performance under diverse environmental conditions, which is essential for commercial adoption.

### 5.2. Selectivity and interference in complex matrices

Although CsPbBr_3_ PNCs exhibit strong halide exchange responsiveness, selectivity in complex chemical environments remains a persistent challenge.^77^ In matrices such as wastewater, sweat, or industrial effluents, the presence of competing anions (*e.g.*, nitrate, sulfate, or organic halides) can partially interfere with the sensor response, leading to signal perturbations or false positives.^78^ Even minimal contamination or pH variations can significantly influence halide exchange kinetics and PL shifts, reducing the reliability of quantitative measurements.

Efforts to enhance selectivity have included ligand engineering, surface functionalization, and encapsulation strategies.^81^ For instance, micellar glycyrrhizic acid capping improves colloidal stability and restricts non-specific interactions, thereby reducing interference from non-halide ions.^84^ Similarly, β-cyclodextrin–arginine networks facilitate selective halide permeation while maintaining high luminescence efficiency.^81^ Nonetheless, these modifications introduce additional synthesis complexity, potentially hindering large-scale production and reproducibility.

Another critical aspect is the influence of ionic strength and co-solvent composition in aqueous samples. High salinity or organic co-solvents can alter the local environment of PNCs, modifying exchange kinetics and fluorescence response.^87^ Thus, achieving consistent and selective sensing in heterogeneous or field-relevant matrices remains an active area of research.

From a practical standpoint, sensor calibration protocols must be matrix-specific, and universal standards are lacking. Advanced approaches, such as machine-learning-assisted signal processing or smartphone-based analysis platforms, may help correct for matrix effects in real time, yet their integration adds complexity and cost to sensor design.^79^ This highlights a key trade-off between operational convenience and analytical fidelity.

### 5.3. Reliability and calibration of wavelength-based chloride sensing

A central advantage of CsPbBr_3_ perovskite nanocrystal sensors is the direct correlation between halide composition and photoluminescence wavelength. During chloride detection, halide exchange between Br^−^ and Cl^−^ modifies the bandgap of the perovskite lattice, producing measurable spectral shifts that enable quantitative calibration. In idealized laboratory conditions, this relationship can often be approximated using Vegard-type compositional trends combined with bandgap bowing models, allowing wavelength shifts to be mapped to analyte concentration.

However, the reliability of wavelength-based calibration in real sensing environments can be affected by several external and intrinsic factors. Temperature fluctuations may induce bandgap shifts independent of halide composition, while variations in pH or ionic strength can alter surface ligand configurations and exchange kinetics.^71,73^ In complex matrices such as environmental water samples or biological fluids, the presence of competing halides or organohalide species may also lead to partial or non-stoichiometric exchange processes. These effects can introduce deviations between measured wavelength shifts and actual chloride concentration.

Instrumental factors further contribute to uncertainty. Differences in spectrometer calibration, detector sensitivity, or smartphone camera spectral response can introduce additional variability when optical readout platforms are used. As a result, quantitative chloride estimation based solely on emission peak position may exhibit measurable error margins under uncontrolled conditions.

To improve reliability, several calibration strategies have been proposed. Multi-point calibration curves constructed under matrix-relevant conditions can reduce systematic bias, while internal reference fluorophores or ratiometric detection schemes can compensate for environmental drift. In addition, emerging approaches based on machine-learning-assisted spectral analysis have shown potential for correcting nonlinearities and matrix effects in real time. In practice, reported uncertainties in wavelength-based chloride sensing typically correspond to spectral deviations of a few nanometers, translating to concentration errors on the order of several percent depending on calibration methodology.^75,76^

Consequently, while wavelength-based sensing remains one of the most powerful features of halide-exchange perovskite sensors, reliable quantitative deployment requires careful calibration protocols that account for environmental conditions, instrumental variability, and long-term material stability.

### 5.4. Industrial and practical applications

The translation of CsPbBr_3_ PNC-based chloride sensors from laboratory prototypes to industrial applications is promising but requires careful consideration of practical constraints.^77^ Rapid, visual colorimetric or photoluminescent detection allows for on-site monitoring of water quality, industrial effluents, and biomedical fluids, with response times ranging from seconds to minutes.^82^ Such features are highly attractive for real-time decision-making in environmental and clinical contexts. However, large-scale deployment necessitates addressing material cost, reproducibility, and stability under operational conditions. While lab-scale syntheses achieve high quantum yields and tunable emission, industrial synthesis must ensure consistent particle size distribution, surface ligand coverage, and photophysical properties across batches.^83^ Variability in these parameters can dramatically influence sensitivity, detection limits, and response linearity.

Integration with portable platforms, including paper strips, cellulose composites, or smartphone-based optical readers, demonstrates considerable potential for field applications.^82,83^ Yet, industrial sensors must also meet mechanical, chemical resistance, and shelf-life requirements, which may not be fully achieved by current designs. Approaches such as cross-linked polymer matrices, hydrophilic capping ligands, or encapsulated composites partially address these limitations, but scalability and long-term performance remain to be validated. Future industrial adoption will also depend on standardization and regulatory approval, especially for applications in potable water monitoring or clinical diagnostics. Strategies that combine low-cost synthesis, high selectivity, and durability will be critical to transform CsPbBr_3_ PNCs from experimental probes to reliable commercial sensors.^83,89^

### 5.5. Integration into portable and real-time sensing devices

The unique optical properties of CsPbBr_3_ PNCs make them well-suited for integration into portable, real-time sensing devices. Smartphone-assisted platforms, handheld fluorimeters, and microfluidic devices have been explored to exploit fast halide exchange-induced L shifts for in-field analysis.^79^ Such integration allows for rapid, quantitative chloride monitoring without the need for centralized laboratories, reducing both response time and operational cost. A critical challenge in device integration is maintaining PNC stability during storage and operation. Water-dispersed PNCs or polymer-encapsulated PNCs demonstrate improved operational lifetimes but may sacrifice sensitivity due to diffusion limitations.^88^ Optimizing the trade-off between accessibility to chloride ions and environmental protection remains a central engineering challenge.

Moreover, the implementation of real-time feedback systems, including automated image analysis or fluorescence peak recognition software, enhances precision but increases device complexity.^77^ Sensor reproducibility across multiple devices and environmental conditions must be rigorously validated before industrial or clinical adoption. Lastly, miniaturization and user-friendliness are critical for end-user acceptance. Devices must combine high sensitivity, rapid response, and intuitive readouts. Innovations such as cellulose-based composites or paper strip sensors demonstrate proof-of-concept solutions but require further optimization, repeatability, and compatibility with existing industrial or clinical workflows.^83^

A realistic route toward commercialization of CsPbBr_3_-based chloride sensors demands compliance with rigorous regulatory frameworks governing lead-containing materials. In the context of consumer electronics and environmental devices, the European Union's REACH (Registration, Evaluation, Authorisation and Restriction of Chemicals) and RoHS (Restriction of Hazardous Substances) directives impose strict restrictions on the amount of lead allowed in any component. Similar principles are enforced in the United States under EPA guidelines for material safety and in biomedical domains under the FDA and EMA directives, where even trace lead exposure can preclude product approval. These frameworks collectively define permissible lead content, disposal standards, and certification pathways that must be integrated into the sensor development process from early design stages to prevent high-cost retrofitting or market rejection.

Meeting these regulations necessitates embedding safety into both materials and product lifecycle. From a materials standpoint, effective encapsulation—using crosslinked polymers, silica matrices, or inorganic shells—ensures mechanical durability and resistance to leaching in operational environments. From a systems viewpoint, the sensors can be incorporated into sealed cartridges or removable units to prevent direct environmental contact. Furthermore, establishing recycling and disposal protocols compliant with hazardous waste management standards is crucial for large-scale deployment. Implementing trackable manufacturing, labeling of lead-containing components, and take-back programs for end-of-life sensors represent practical steps aligning commercialization with environmental responsibility. Proactive adoption of these strategies will position perovskite-based technologies on a realistic and legally compliant path toward market introduction.

### 5.6. Environmental, health, and regulatory considerations

The presence of lead in CsPbBr_3_ PNCs poses environmental and health risks that must be considered for industrial or widespread deployment.^84^ Lead leakage during synthesis, disposal, or accidental spillage can introduce toxic hazards, necessitating proper containment, recycling, and regulatory compliance. Current research emphasizes encapsulation or polymer matrices not only for stability but also for reducing lead bioavailability.^88^ Regulatory frameworks for heavy-metal-containing sensors vary by region. For example, water quality monitoring devices may require certification to ensure that sensor materials do not contribute to contamination, while biomedical applications face stringent clinical safety standards.^89^ Early consideration of these requirements is critical for successful commercialization.

From a sustainability perspective, the development of lead-free or reduced-lead perovskite alternatives is an active area of research. Mixed halide systems or tin-based analogues may offer comparable photophysical performance while mitigating environmental concerns, although they often present new stability or sensitivity challenges.^84^ Finally, lifecycle analysis and end-of-life disposal strategies will influence the practical adoption of CsPbBr_3_-based sensors. Industrial-scale implementation must balance sensor performance with environmental responsibility, ensuring that the benefits of rapid, real-time chloride detection are not offset by long-term ecological or health risks.

Understanding and minimizing lead leakage under real use conditions is essential for risk assessment and public health acceptance. Standardized testing protocols—based on accelerated aging, humidity cycling, and pH stress—should be employed to simulate realistic sensor lifespans and mechanical stresses. These tests can unveil encapsulation fatigue or microcrack formation that may permit lead ion escape over extended use. Complementary toxicological characterization in simulated biological and environmental media can determine the bioavailability of released ions and their potential uptake. Establishing quantitative correlations between measured leakage rates and physical degradation mechanisms provides critical data for validating sensor safety claims and satisfying ALARA (As Low As Reasonably Achievable) criteria under regulatory scrutiny.

Beyond containment, addressing Pb toxicity also requires a forward-thinking materials roadmap. Hybrid or lead-free alternatives, such as Sn-, Bi-, or double perovskite compositions, continue to evolve but have not yet matched CsPbBr_3_ in stability or photoluminescent response. A transitional strategy can thus focus on maximum containment of lead-based sensors for controlled environments coupled with parallel development of lead-free analogues for wider applications. Innovations such as self-healing encapsulation layers or “leak sensors” that visibly signal barrier failure could further assure safe usage during the lifetime of these devices. These combined engineering and material-level strategies create a bridge toward biosafe perovskite sensing systems that align with both environmental protection and long-term sustainability.

### 5.7. Lead leakage, biosafety, and long-term environmental impact

Despite their attractive sensing performance, CsPbBr_3_ nanocrystals inherently raise concerns related to lead toxicity, particularly in biological and wearable sensing applications. Potential lead leakage may arise from partial lattice degradation, surface dissolution under prolonged exposure to aqueous or biological media, or mechanical damage to encapsulation layers. Even trace levels of bioavailable Pb^2+^ pose significant risks in physiological environments, necessitating strict mitigation strategies.

Encapsulation within polymeric matrices, mesoporous oxides, or hybrid composites has been shown to substantially reduce lead release by physically confining the perovskite and limiting direct contact with biological fluids. Importantly, such strategies reduce bioavailability rather than eliminating lead entirely, and their long-term integrity under continuous mechanical stress or enzymatic exposure remains insufficiently explored. For wearable or implantable formats, even slow cumulative release over extended operation times could become problematic.

From an environmental perspective, disposal and end-of-life handling of perovskite-based sensors represent additional challenges. Without controlled recycling or containment, damaged or discarded devices may contribute to localized lead contamination. These considerations are particularly relevant for large-scale deployment in water monitoring or disposable test strips.

As a result, the use of CsPbBr_3_-based sensors in biological contexts should be approached with caution and limited to well-encapsulated, non-invasive formats unless rigorous toxicological validation is performed. Parallel development of lead-reduced or lead-free perovskite analogues, while maintaining halide-exchange-driven optical tunability, represents a critical future direction to reconcile sensing performance with environmental and health responsibility.

### 5.8. Grand challenges and future research directions

Despite the significant advances achieved in CsPbBr_3_ perovskite nanocrystal-based chloride sensing, several key challenges must be addressed before these systems can reach widespread practical deployment. Addressing these challenges will require coordinated efforts spanning materials science, analytical chemistry, device engineering, and environmental safety assessment.

One of the primary challenges lies in achieving intrinsic material stability. Although encapsulation strategies have improved environmental tolerance, many current approaches rely on external protection layers that may limit ion accessibility and reduce sensing responsiveness. The development of intrinsically stable perovskite nanocrystals with improved resistance to moisture, oxygen, and thermal fluctuations remains a critical research objective.

Another major challenge involves improving selectivity and quantitative reliability in complex matrices. Environmental and biological samples often contain multiple competing ions and organic species that can influence halide exchange kinetics or photoluminescence behavior. Advanced surface functionalization strategies, selective ligand engineering, and machine-learning-assisted signal processing may help improve sensor robustness and accuracy under realistic operating conditions.

Scalability and manufacturing reproducibility also represent important barriers to commercialization. Industrial production of perovskite nanocrystals must ensure consistent particle size distribution, ligand coverage, and optical properties across large batches. Developing low-cost, scalable synthesis routes while maintaining high photoluminescence efficiency and sensing performance will be essential for translating laboratory demonstrations into deployable technologies.

Environmental and health considerations will also shape the future development of perovskite-based sensors. The presence of lead in CsPbBr_3_ nanocrystals necessitates continued efforts toward improved encapsulation, safer device architectures, and the exploration of lead-reduced or lead-free perovskite analogues that retain halide-exchange responsiveness.

Finally, future sensing platforms are likely to benefit from integration with portable electronics, microfluidic systems, and digital data-processing tools. Smartphone-assisted analysis, automated spectral interpretation, and Internet-of-Things (IoT) connectivity could enable real-time monitoring of chloride concentrations in environmental, industrial, and biomedical settings. These developments may ultimately transform perovskite nanocrystal sensors from laboratory curiosities into versatile tools for decentralized chemical monitoring.

Addressing these grand challenges will not only improve the reliability and safety of CsPbBr_3_-based sensors but also broaden their potential applications across environmental monitoring, healthcare diagnostics, and industrial process control.

## 6. Conclusion

CsPbBr_3_ PNCs have emerged as highly promising luminescent probes for rapid, sensitive, and selective chloride ion detection across diverse matrices, ranging from industrial wastewater to biological fluids. Their unique halide exchange-induced PL shifts enable real-time monitoring with minimal sample preparation, offering advantages over conventional analytical methods in speed, portability, and visual accessibility. Mechanistic insights reveal that the high ionic mobility within the perovskite lattice, coupled with surface ligand engineering, underpins both the sensitivity and tunability of these nanosensors. Despite these strengths, practical deployment is challenged by inherent instability under moisture, heat, and polar solvents, potential interference from coexisting ions, and environmental concerns due to lead content. Advances in ligand engineering, encapsulation, and polymer or cellulose-based integration have mitigated some limitations, enhancing photostability, selectivity, and compatibility with portable sensing platforms. Furthermore, smartphone-assisted detection and visual colorimetric approaches have expanded the applicability of CsPbBr_3_ PNCs in both industrial and biomedical contexts, bridging the gap between laboratory research and field implementation.Looking forward, future development should focus on scalable synthesis of stable PNCs, reduction of lead toxicity, and standardization for real-world industrial or clinical use. Innovations in mixed-halide or lead-free perovskites, coupled with machine-learning-assisted signal processing, may further enhance reliability, reproducibility, and environmental sustainability. Overall, CsPbBr_3_ PNC-based chloride sensors represent a versatile and transformative class of nanomaterials, poised to redefine rapid anion monitoring in analytical chemistry, environmental surveillance, and healthcare diagnostics.

## Conflicts of interest

The authors declare that they have no known competing financial interests or personal relationships that could have appeared to influence the work reported in this paper.

## Data Availability

This article is a review and does not include any new experimental data. All data discussed and analyzed are derived from previously published studies, which are appropriately cited in the manuscript. No new datasets were generated or analyzed during the current study.
